# Cost-Effective Smartphone-Based Articulable Endoscope Systems for Developing Countries: Instrument Validation Study

**DOI:** 10.2196/17057

**Published:** 2020-09-10

**Authors:** Youngjin Moon, Jeongmin Oh, Jaeho Hyun, Youngkyu Kim, Jaesoon Choi, Jeongman Namgoong, Jun Ki Kim

**Affiliations:** 1 Biomedical Engineering Research Center Asan Institute for Life Sciences Asan Medical Center Seoul Republic of Korea; 2 Department of Convergence Medicine College of Medicine University of Ulsan Seoul Republic of Korea; 3 Department of Biomedical Engineering College of Medicine University of Ulsan Seoul Republic of Korea; 4 Department of Surgery Asan Medical Center Seoul Republic of Korea

**Keywords:** smartphone-based endoscope, mobile health, low-resource settings, continuum body, articulable endoscope, low-cost medical device, point of care diagnostics

## Abstract

**Background:**

Endoscopes are widely used for visualizing the respiratory tract, urinary tract, uterus, and gastrointestinal tracts. Despite high demand, people in underdeveloped and developing countries cannot obtain proper access to endoscopy. Moreover, commercially available endoscopes are mostly nonarticulable as well as not actively controlled, limiting their use. Articulating endoscopes are required for some diagnosis procedures, due to their ability to image wide areas of internal organs. Furthermore, actively controlled articulating endoscopes are less likely to harm the lumen than rigid endoscopes because they can avoid contact with endothelial tissues.

**Objective:**

The study aimed to demonstrate the feasibility and acceptability of smartphone-based wide-field articulable endoscope system for minimally invasive clinical applications in developing and less developed countries.

**Methods:**

A thin articulable endoscope system that can be attached to and actively controlled by a smartphone was designed and constructed. The system consists of a flexible endoscopic probe with a continuum mechanism, 4 motor modules for articulation, a microprocessor for controlling the motor with a smartphone, and a homebuilt app for streaming, capturing, adjusting images and video, and controlling the motor module with a joystick-like user interface. The smartphone and motor module are connected via an integrated C-type On-The-Go (OTG) USB hub.

**Results:**

We tested the device in several human-organ phantoms to evaluate the usability and utility of the smartphone-based articulating endoscope system. The resolution (960 × 720 pixels) of the device was found to be acceptable for medical diagnosis. The maximum bending angle of 110° was designed. The distance from the base of the articulating module to the tip of the endoscope was 45 mm. The angle of the virtual arc was 40.0°, for a curvature of 0.013. The finest articulation resolution was 8.9°. The articulating module succeeded in imaging all 8 octants of a spherical target, as well as all 4 quadrants of the indices marked in human phantoms.

**Conclusions:**

The portable wide-field endoscope was successfully controlled using a smartphone, yielding clear images with a resolution of 960 × 720 pixels at realistic focal distances. Actively and precisely controlled articulating movements have resulted in minimally invasive monitoring in the narrow space of internal organs providing a wide-area view. We found our smartphone-based active articulated endoscope to be suitable for point-of-care applications in developing and less developed countries.

## Introduction

Over the past few decades, endoscopy has become a fundamental clinical approach for noninvasive screening and diagnosis of various diseases. Now, endoscopy designers have expanded the range of functions to include longitudinal observation in noninvasive methods, simple medical procedures, biopsy, local therapy, or minimally invasive robotic surgery [1,2]. Despite their essential utilities for screening and diagnosis, clinical endoscopes rarely leave the hospital; thus, they are used mainly for clinical applications such as biopsy and staging for diseases.

In resource-poor undeveloped and developing countries, it is difficult to obtain proper endoscopy service. For example, the majority of hospitals in Nigeria have no facilities for endoscopy [3]. Moreover, less developed countries often have serious power supply problems. The medical power supply does not satisfy the needs of patients; consequently, proper medical care relies on local generators. The poor supply of power has deleterious effects on medical endoscopy, because endoscopic procedures are abruptly stopped due to power interruptions [3]. In addition to power deficiencies, several other problems in developing countries disrupt endoscopy service to serious levels. The limited number of hospitals that are able to provide endoscopy services, poor maintenance, and broken endoscopes, as well as the long distances between rural areas and large hospitals, combine to prevent people in need from receiving proper endoscopy service.

Despite the increasing demands for overcoming current problems, there have been few attempts to integrate medical devices into smartphones having ample potential for solving those limitations. In addition to having the performance of a personal computer, smartphones are portable and can be equipped with many useful peripherals, including selfie sticks, USB devices, sensors, and supplementary microprocessors. However, most of the apps of smartphone devices are based on the sensors built into smartphones, such as the cameras, accelerometer, gyroscope, magnetometer, and barometer. Those functions are commonly used to determine the user’s position, movement, and posture. Some of the smartphone attachments are developed for biological analysis with simple optical devices which enable to perform enzyme-linked immunosorbent assay analysis [4] and immunoassays or to collect and analyze recombinant bovine somatotropin microsphere assay data of food allergens [5].

There are various smartphone-based endoscopes on the market. These endoscopes are small, long webcams that can be connected to smartphones via USBs that enable endoscopic webcams to be connected to desktops, laptops, and even smartphones [6]. The price range of these peripheral devices varies from US $10 to $100 [7]. These peripheral devices are employed for industrial applications such as pipe and car inspection. They are mostly flexible to various degrees, including flexible rubber covering inner electronics and a long spine-like exoskeleton that can be fixed in a position when shaped by external forces; however, the flexible probe cannot be actively controlled. These passively bendable endoscopes have a long, stiff top-head and large diameter due to their exoskeletal rubber jacket, making it difficult to access narrow spaces. Endoscopes with a spine-like exoskeleton are not only extremely thick to be employed for medical examination but also extremely rigid, thus presenting a risk of harming the soft inner surface of the organs [8]. Therefore, neither of these endoscopes are suitable for medical inspection. Moreover, none of the commercially available smartphone endoscopes are actively articulable; thus, they cannot actively scan tubular organ canals. A few rigid endoscopes are designed such that they can inspect the insides of the human body; however, they are rigid and short, limiting them to the shallow parts of the body (eg, the nostrils and entrance to the ear canals).

The articulating endoscope is needed in medical diagnosis for several reasons. The first is patient safety and comport. Patient comfort following a minimally invasive procedure differs significantly with flexible versus rigid endoscope use. In addition, actively articulated flexible endoscopes can provide better safety and comfort than passively articulated ones. Gmeiner et al [9] reported that flexible endoscopy resulted in more patients being comfortable after treatment, while rigid endoscopy always required general anesthesia or sedation during the procedure. For example, not all patients tolerate rigid laryngoscopy, particularly those with a sensitive gag reflex or limited jaw or neck mobility [10]. Sensitive patients or patients with limited movements undergoing rigid laryngoscopy are more likely to feel uncomfortable during and after the procedure. Additionally, patients reported less pain during cystoscopy performed with flexible cystoscopes compared with rigid cystoscopes [11,12]. The second reason why articulating endoscopes are needed in medical diagnosis is that they can provide wide-area monitoring. Since the intestinal canal or cavities in the human body have mostly abrupt bending and curves, the rigidity of the endoscope significantly influences the range of view that the medical professional can observe in the organ. Unlike rigid endoscopes, articulating endoscopes achieved an imaging success rate of 90% and covered almost all ranges and directions as long as they had proper bending angles [13,14].

In this paper, we present a design to satisfy the need for an actively controlled articulating endoscope with smartphone connectivity and mobile medical device applications, in the absence of commercially available equivalent. Our device connects to a smartphone via USB connection, and a homebuilt app allows the user to successfully adjust images, control the endoscope’s articulable robotic body, and capture still images and movies for later analysis. With a supplemental microprocessor, the articulable robotic continuum body achieves motion steps of 8.9°, and the camera offers a resolution of 960 × 720 pixels at 30 fps and a depth of field of 10 to 80 mm. For additional portability, the device may be powered entirely by the smartphone’s USB port, or supplementary batteries may be used. The designed device is easily cleanable and self-illuminated and attains a wide field of view and suitable resolution for clinical imaging in phantoms of the human larynx and bladder. We hope that it will provide a strong development base to satisfy clinical needs in underdeveloped countries.

## Methods

### USB-Integrated Endoscope for Smartphone Appendage

The articulating and portable endoscope device was designed for the smartphone by integrating 8 parts: smartphone, four servomotors, microprocessor, articulating continuum robot tip module, wire-based system for tip articulation, USB connection circuit, case for smartphone mounting, and commercially available industrial endoscope module. The endoscope module (3.9 mm-diameter medical endoscope module with a USB interface, 100° large field of view model, Shanghai Chenyu Smart Technology Co, Ltd) supports USB 3.0 connection, features light-emitting diode illumination, and is compatible with Intel USB chips. With a diameter of 3.9 mm and a head length of 10.6 mm, the USB endoscope module is smaller in diameter than commercially available industrial endoscopes, and its thickness and length are suitable for navigating and imaging the esophagus, larynx [15], and bladder [16], which are frequently examined. The complementary metal-oxide semiconductor (CMOS) sensor of the endoscope module uses the ov9734 chip from Omnivision, which supports a resolution of 960 × 720 pixels, 30 fps video streaming, LED light source, and programmatic adjustment of the brightness, contrast, white balance, and exposure. The depth of field is 10 to 80 mm, which allows a large range of imaging in internal organs.

**Figure 1:**
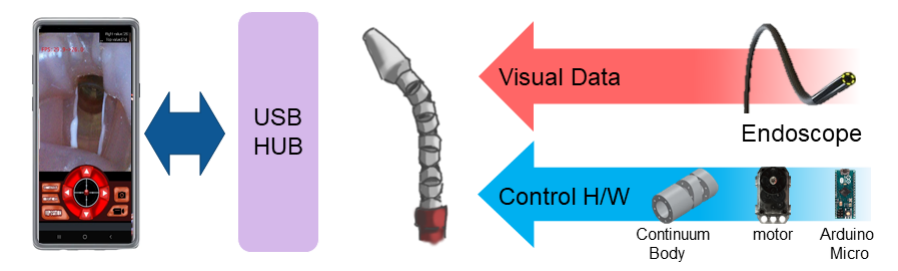
Overall schematic of the data flow of the articulating mobile endoscope. The smartphone is attached to the articulating module, which is a combination of the endoscope module and the continuum body.

A general schematic of the endoscope is shown in Figure 1. The app functions as a software platform for the camera and for control of the snake-like continuum body. The devices are connected by an on-the-go (OTG) USB connection. USB devices intrinsically have a master/slave relationship. Only the master device can control a slave device. Smartphones are typically considered to be slave USB devices. Therefore, if connected normally, a smartphone cannot control the attached cameras and send a signal to a microprocessor. However, with the OTG USB hub connection, the roles of the master and slave can easily be changed, allowing the smartphone to serve as the host to peripheral devices [17]. A microprocessor and camera unit serve as the slave units, while the smartphone acts as a host.

**Figure 2:**
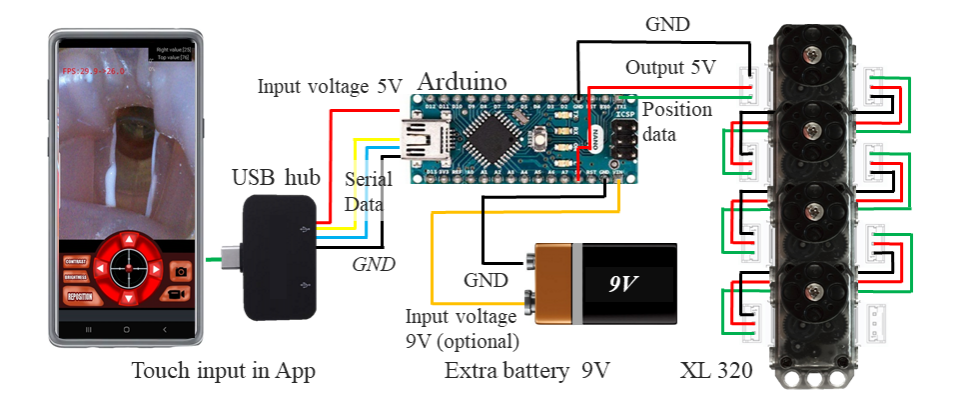
Electric control diagram of the articulation control system. The touch input from a smartphone app is processed into motor control signals using an Arduino processor. The extra battery is optional.

Through the OTG USB hub connection, the app, endoscope CMOS module, and microprocessor can interact with each other. Figure 2 presents a simplified circuit diagram of the device. With the OTG USB hub connection secured, the app obtains permission to access the camera and microprocessor. We used the Arduino Nano as a microprocessor, which is an open-source electronics platform, so that it can be easily used for development and circuit building [18]. The Arduino, motor, and smartphone all have the same 5V operating voltage. Extra batteries can be connected to the device if necessary, but primary electricity is supplied by the smartphone’s lithium-ion battery. The OTG USB hub connection also functions as an electricity-providing hub.

The microprocessor is connected to 4 motors, which are connected to wire-controlling pulleys. The motor (Dynamixel XL-320, Robotis) is a robot-exclusive smart actuator with a fully integrated direct current motor, reduction gearhead, controller, driver, and network in one direct current servo module [19]. Each motor has its own motor driver, allowing intuitive control by the microprocessor software.

**Figure 3:**
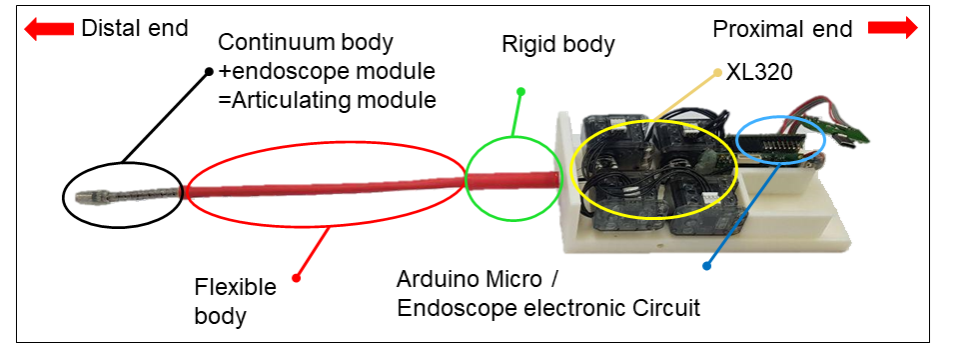
Cut-away assembly of the device, showing the actual structure. The long flexible distal end is composed of the continuum, flexible, and rigid bodies.

Figure 3 depicts the assembled device prototype, complete with the 3-dimensional (3D)–printed case. The motor, microprocessor, and OTG USB hub are assembled within a proximal case. From the case, the rigid segment connected to the flexible segment protrudes. At the most distal part, the continuum robot is assembled around the endoscope to form the articulating module. Four motors are affixed to the bottom of the base of the case.

### Continuum Body

**Figure 4:**
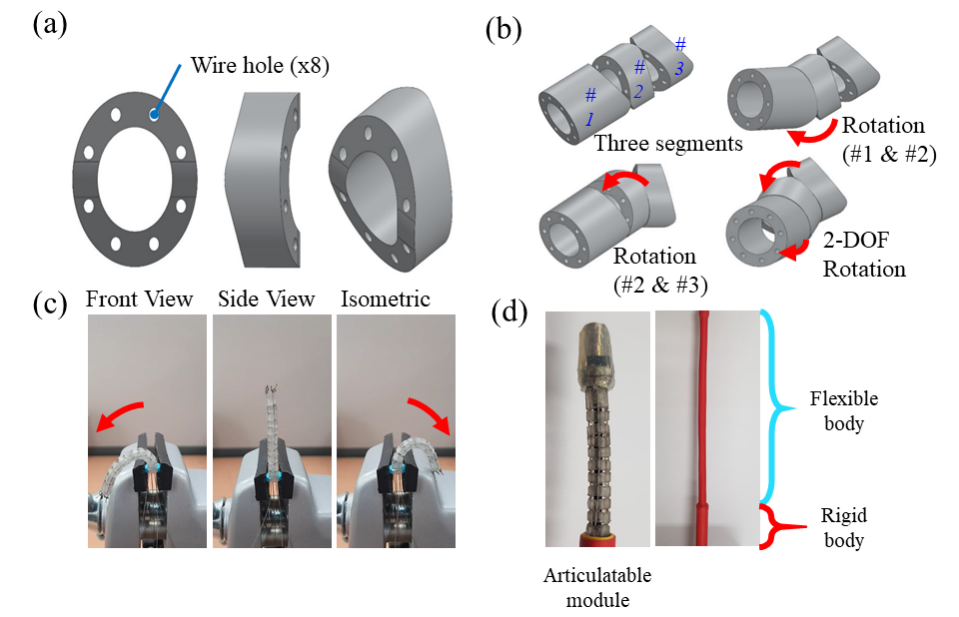
Structure of the continuum body: (a) 3D model of an individual unit of the continuum mechanism, (b) movement combination of three individual segments, resulting in 2-degree of freedom rotation, (c) bending motion of the 3D-printed continuum body prototype, and (d) prototype of the flexible and rigid bodies.

The continuum body is designed to have a snake-like motion. This structure comprises several small segments connected via wires. An individual segment in the continuum body is portrayed in Figure 4A. The ring-like individual segment with 8 wire holes has internal space that can be used for essential internal components (ie, cameras); further, this space helps reduce the weight of the segment and allows for easy maintenance. The two individual segments shown in Figure 4A comprise rolling joints, and their protruding surfaces are in contact. Figure 4B shows the simulated articulation of 3 individual segments (#1,2,3) connected by control wires, using which a combination of multiple segments can move with various degrees of freedom (DOFs) [20]. Such a multijoint tendon-driven mechanism is common in medical devices because of its efficiency, robustness, and reliability [21]. The combination of the segments is articulated by applying forces to pull the control wires, which torque to the final segments about the slopes on the connecting edges [22]. The rotation axis formed by the slopes between segments #1 and #2 is perpendicular to that formed by segments #2 and #3. Therefore, a combination of 3 segments can make the arbitrary orientation of the distal segment in 2 DOFs, as shown in Figure 4B.

Figure 4C shows the bending of a 3D-printed continuum body prototype. The bending angle is sufficient to make the tip of the continuum body face the proximal direction. When a user touches a controlling joystick-like user interface (UI) in a smartphone app, the microprocessor controls 4 motors to tighten and loosen the pair of wires. As shown in Figure 4B, this gives the continuum mechanism 2 DOFs, which provides full freedom in movement within a 2D plane [23]. Therefore, with adjustment of the tension, the continuum mechanism can navigate through complex spatial obstacles. The model of each continuum body backbone was designed using SolidWorks 2018 (Dassault Systèmes SolidWorks Corporation) and then printed by a 3D printer.

Figure 4D shows a completed articulating module prototype, assembled with flexible and rigid bodies. The articulating module is combined with the flexible and rigid bodies to adjust the distance from a target to the controller case. The flexible body is approximately 15 cm long, allowing endoscope to smoothly slither into an organ. The rigidity of the flexible bodies is selected to suit its role. It is flexible enough to pass through canals, similar to a traditional flexible endoscope, and rigid enough for wires not to bend this part during articulation. The rigid body forms the base of the articulating wire system and is approximately 5.5 cm long.

**Figure 5:**
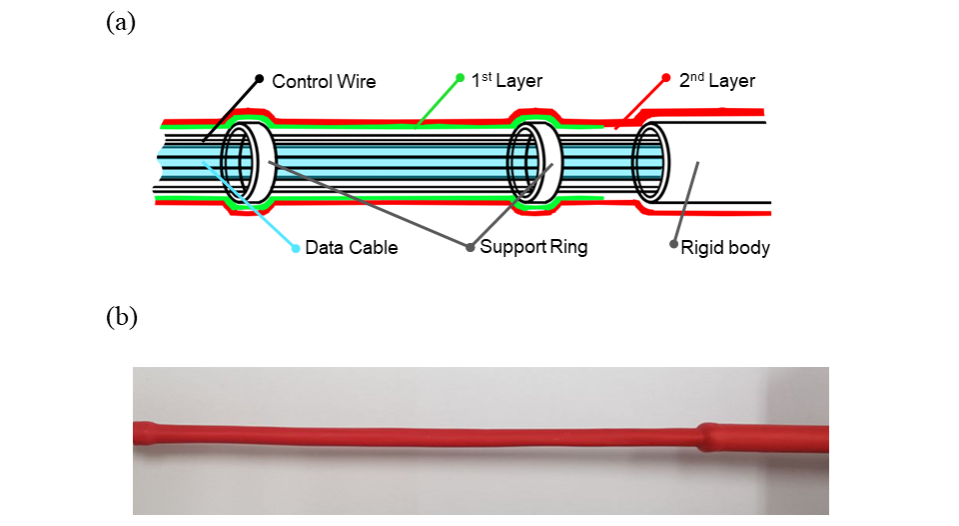
Flexible segment of the endoscope module: (a) structure of the flexible segment, designed to flex to the curvature of the organ and also be stiff enough to relay the tension of the controlling wires to the articulating module and (b) fabricated flexible segment.

The flexible segment and rigid segment were jacketed with a waterproof material, polyolefin, as shown in Figure 5. The jacketing process is essential for an endoscope, to protect the inner electronics from the humidity of the organ lumen and protect the patient from mechanical debris. As shown in Figure 4, the assembled segments have lateral gaps between segments. These gaps may allow mucus or substances found on the interior of an organ to enter the central hole of the articulating module, leading to circuit failure as well as injury to the vulnerable endothelium from clipping when the lateral gap diminishes.

To prevent the flexible segment from being tensioned by the continuum segment, we applied double jacketing and added support rings for the flexible segment. Figure 5A depicts the internal structures of the flexible and rigid segments. The data cable in the center is surrounded by wires and relays tension to the continuum segment. The control wires pass through holes in the support rings. The support rings perform two major functions: preventing 8 wires from tangling in the middle of the flexible segment and allowing the flexible segment to relay sufficient force to the continuum segment, making the endoscope articulable and more responsive to the control.

While the first jacketing layer extends to the support rings, the second layer thoroughly covers the entire outer surface of the flexible segment. To ensure transparency for the articulating module, the continuum segment is jacketed in transparent latex. With double jacketing, the sheath became thick enough for the flexible segment to resist the torsional forces of the continuum segment, while remaining thin enough to fit into the sharp curvature of internal body parts. When jacketing was complete, these lateral gaps were covered by the sheath. We used a contractile tube for the sheathing and verified its feasibility in a phantom application. For clinical applications, the sheathing can be replaced with materials that are more biocompatible.

### Controller Case

**Figure 6:**
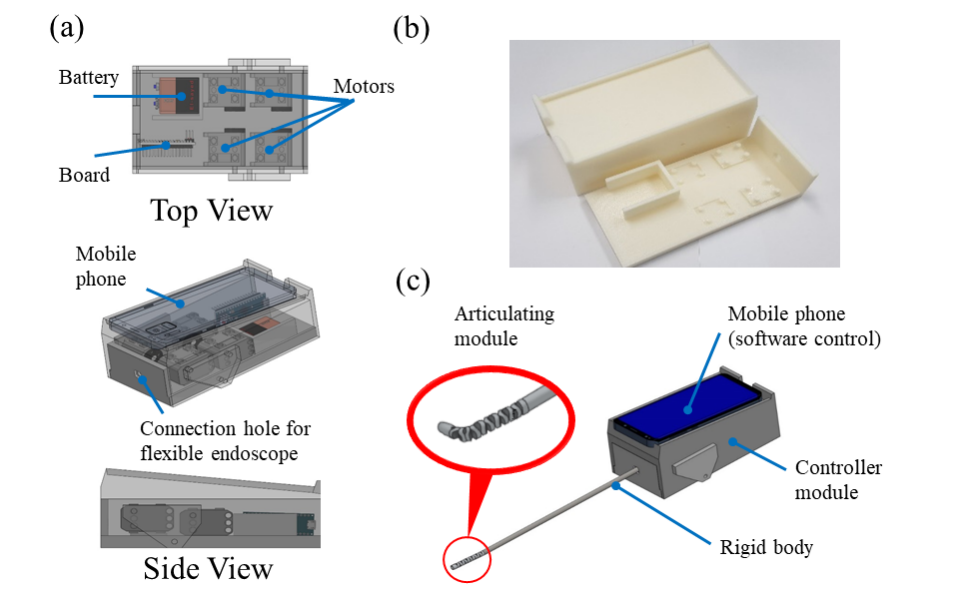
Controller box: (a) configuration of the components in the controller box, (b) 3D printed case of the controller box (base of case immobilizes the motors, and top lid serves as mount for the smartphone), and (c) overall outer blueprint of assembled case and magnified image of the articulating module.

The controller case comprises a lid and a base, including motors and electronic boards, as shown in Figure 6. Four wires controlling the articulating module are bound to the pulleys with projecting screws installed on the rotary shafts of the respective motors. The base part is designed so that a pair of motors are mounted vertically at different heights to ensure that they are connected to each pulley without tangling from the connection hole. The remaining space of the controller case is reserved for the battery, control board, board for signal connection, etc. The lid part is designed so that a smartphone can be placed on the part, and the rear side of the case—opposite to the articulating module—is formed in an open structure so that the signal line from the controller case can be easily connected to the smartphone inlet on the upper part. The model of the case was designed using SolidWorks 2018, and the prototype was constructed via 3D printing. The dimensions of the control case are 150 mm (length), 76 mm (width), and 65 mm (height). The fabricated devices can be equipped with a 5V battery (9V extra) and a 140 mm (length) by 70 mm (width) size smartphone. The price for hardware construction are presented in Table 1.

**Table 1:** Hardware prices of the articulable endoscope device.

Hardware	Price US$
XL320	22
3.9-mm camera module	170
3D printing of case	16
3D printing of continuum	212
Controller	19
Wires, etc.	34
Total price	473

## Results

### User Interface and Algorithm for Smartphone App

We developed an app written in the languages Java and C to provide a straightforward and intuitive interface. The Java app included the UVC camera library developed by saki4510t [24] to enable the user to use the USB camera, as well as the USB serial library [25] for serial communication between smartphone and microprocessor. The virtual joystick library developed by Damien Brun [26] and directional buttons are employed to enable intuitive motor control.

**Figure 7:**
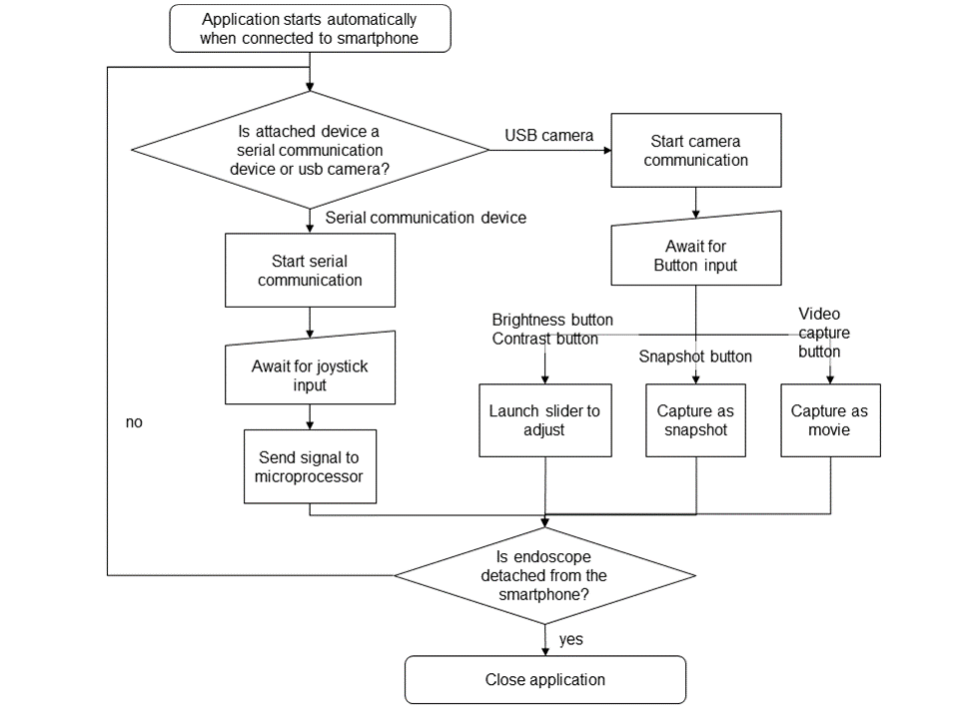
Overall workflow algorithm of the app.

Figure 7 describes the general workflow algorithm of the app. The app automatically launches when the device is attached to the smartphone. When the OTG USB hub connection of the two device components (endoscope module and microprocessor) is secured, the app automatically distinguishes the two components of the device; one is the serial communication device (ie, the microprocessor) and the other is the CMOS chip of the endoscope camera. These two simultaneous threads allow the app to display image data from the camera while sending signals to the microprocessor. When the communication thread is launched, the app opens the intuitive UI, where the user can control the camera and motor simultaneously.

**Figure 8:**
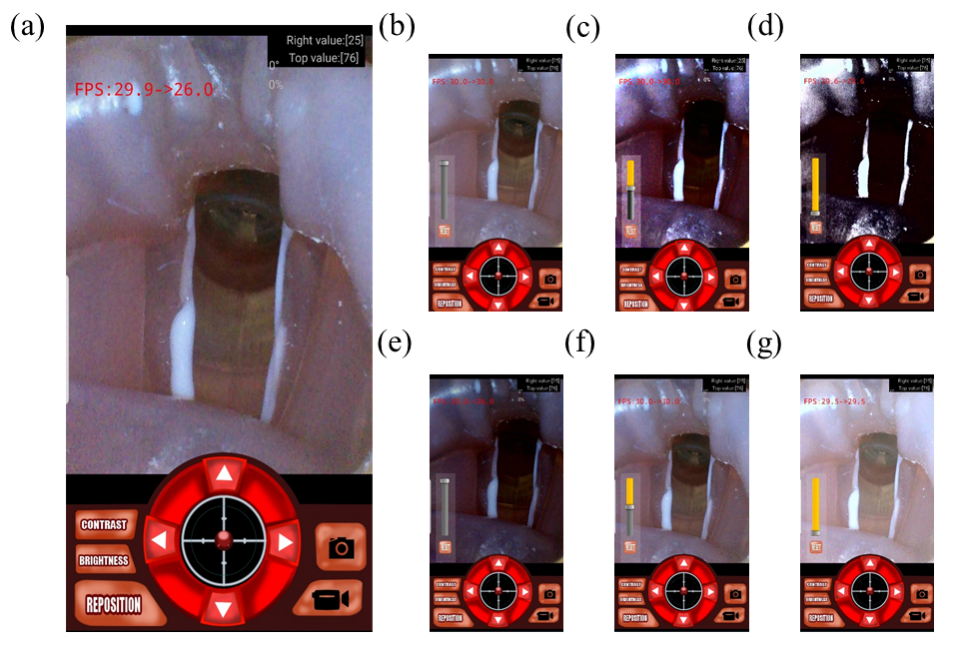
Software-controlling user interface (UI) of the homebuilt smartphone app: (a) main UI of the app (joystick and motor control buttons are in the center, contrast and brightness adjustment buttons are on the left, and movie and still-image capture buttons are on the right), (b-d) slider UI for contrast adjustment and corresponding processed images, and (e-g) slider UI for brightness adjustment and corresponding processed images.

The UI of the app is presented in Figure 8A. The app functions as a viewer, controller for the camera, motor controller, and recording device. A large center image viewer relays visual signals received by the CMOS chip in real time. The virtual joystick UI allows the user to intuitively control the motors by holding it in position, similar to the joystick of a game controller. If the user touches and drags the joystick-like controller, the app sends calculated intensity signals to the microprocessor. The microprocessor winds motors to induce movements corresponding to the virtual joystick. When the user releases the joystick-like controller, the red-dot pointer annotating the figure position returns to the origin point, and the motors stop. The 4 directional buttons are arranged around the joystick-like controller. The buttons function as a secondary motor controller, allowing the user fine control over the articulation of the endoscope. The reposition button is located on the lower left of the UI and makes the articulating module return to its origin point.

The app allows the user to capture the current view in two formats: as a still image and as a movie. The still capture button and video capture button at the bottom right of the screen perform this task. Image and videos are stored automatically in separate folders.

We arranged the button and slider UI to provide control over brightness and contrast to the user. Figures 8B-D depict the contrast adjustment slider and corresponding changes in images. If the user touches the contrast button on the bottom left of the screen, the semitransparent slider bar with the slider bar reset button appears on the screen. The user can easily adjust the contrast by touching the slider UI. The reset button resets the slider parameter to the default setting. Brightness adjustment is performed similarly. Figures 8E-G depict the brightness adjustment slider and corresponding image transformation.

### Articulation Test

**Figure 9:**
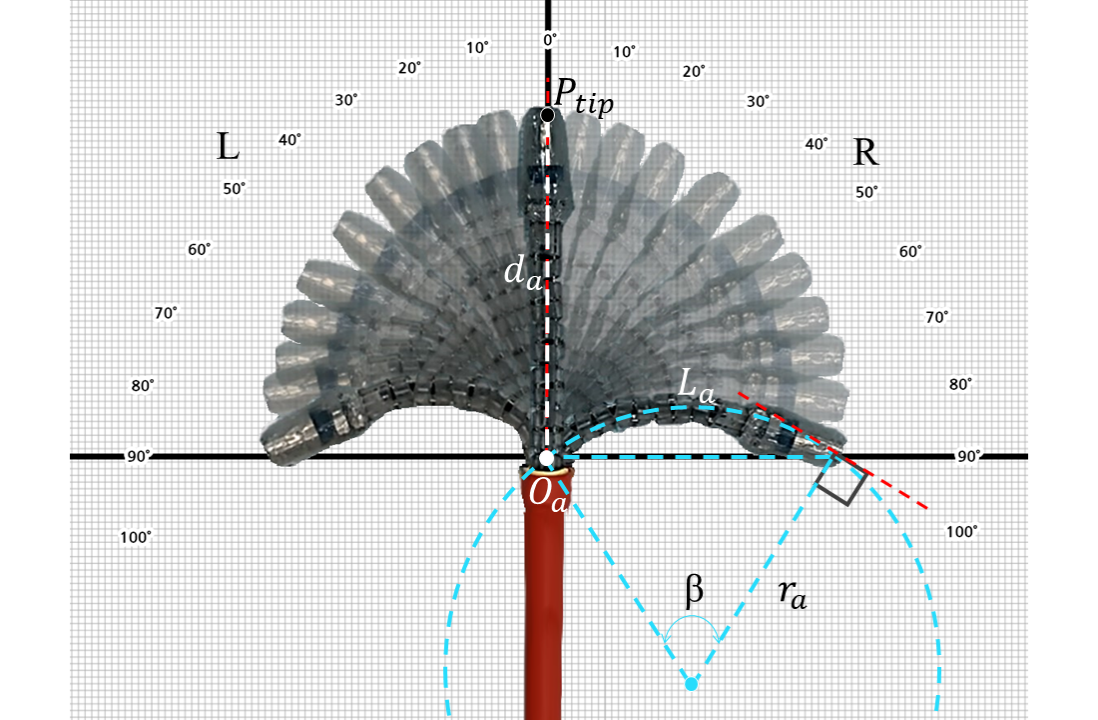
Experimental composite image showing the articulating range of the articulating module. The radius of curvature reaches 74.5 mm, and angle extends to 100°.

A bending characteristics experiment was conducted to evaluate the motion and imaging ability of the designed endoscope device. In the planar bending experiment, the articulating module of the device was bent atop plotting paper, as depicted in Figure 9. Although the theoretical maximum bending angle was 177°, the maximum bending angle was set as 110° for structural safety of the articulating module, as depicted in Figure 9. The distance dα from the origin Oα to the tip Ptip was 52 mm for a collinear position, which was defined as a bending angle of 0°, and dα was 45 mm for the maximum bending angle. The virtual arc for maximum bending can be expressed as Lα=rαβ, where Lα is dα in the collinear position, rα represents the radius of the arc, and β represents the angle of the arc. The curvature κ is simply calculated via the inversion of rα. In the experiment, β was approximately 0.698 rad (40.0°), rα was approximately 74.5 mm, and κ was approximately 0.013. These values can be adjusted by designing the joint angle between the two segments of the continuum body.

**Figure 10:**
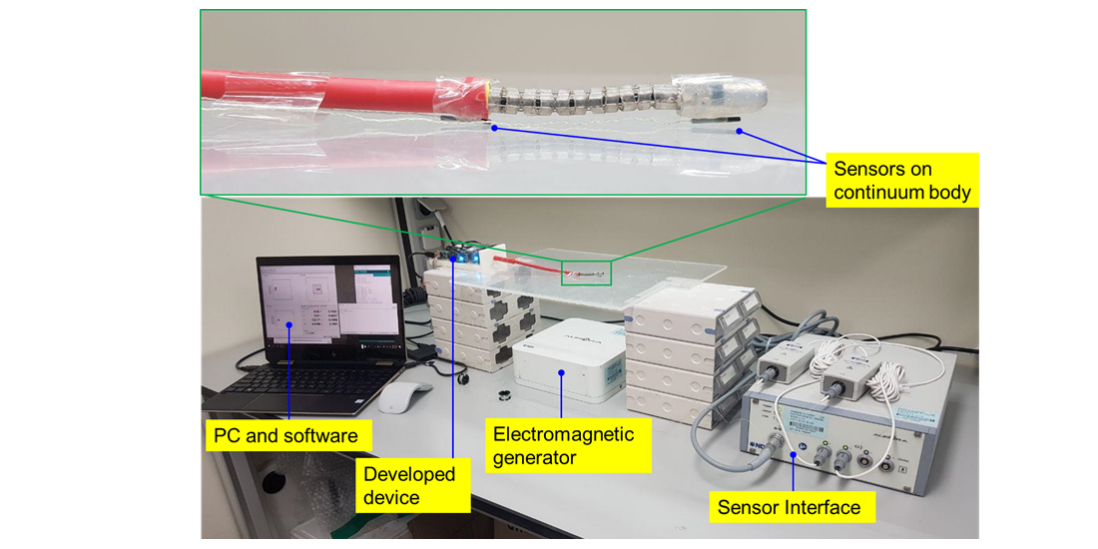
Sensor interface setup for the planar bending test.

**Figure 11:**
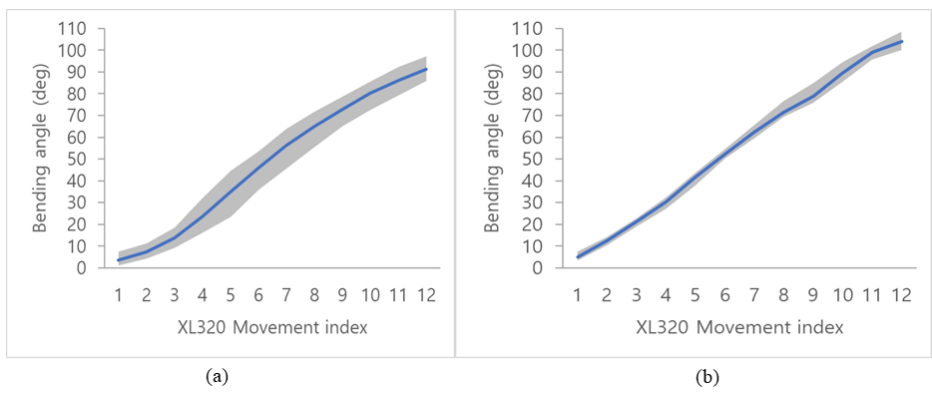
Relationship between the control input and the bending angle in the (a) right and (b) left directions.

Meanwhile, the planar bidirectional bending for unit input movement of the motor was tested 25 times for each direction as shown in Figure 10; the resulting angles are plotted in Figure 11. In the experiment, the position of the tip of the continuum body was measured using an electromagnetic tracking system (Aurora System, Northern Digital Inc), and the setup is shown in Figure 10. Using the aforementioned smartphone app, the incremental input of the motor XL320 was varied by 8.9° per movement index. In Figure 11, the blue lines and gray areas denote averages and variances, respectively; further, a close trend of linear correlation is illustrated. The result demonstrate maximum standard deviations of 5.7° and 2.2° for right and left bending, respectively.

**Figure 12:**
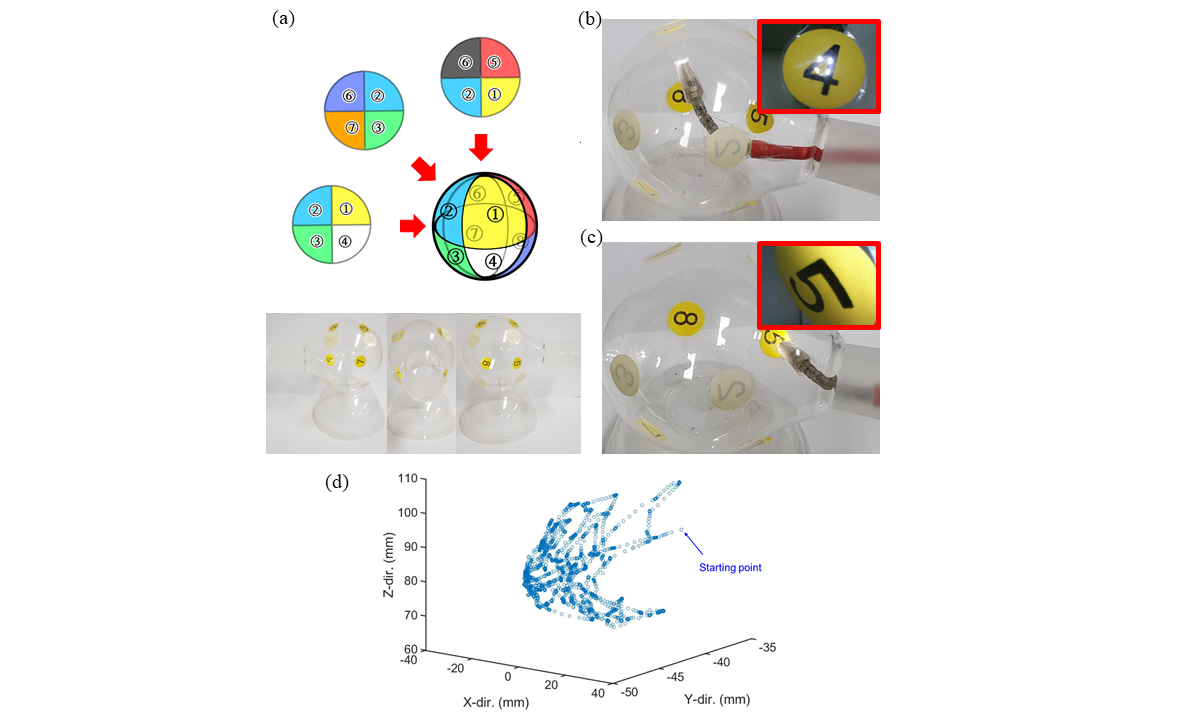
Spatial bending test of the continuum body: (a) test sphere divided into eight regions, (b) facing region ④ (inset: endoscopic view of region ④), (c) approaching region ⑤ (inset: endoscopic view of region ⑤), and (d) spatial trajectory of a trial.

In the spatial bending experiment, the spatial bending motion and image for randomly selected directions were verified by visualizing the insides of the transparent sphere octant. This surface (with a diameter of 85 mm) was prepared and divided into 8 sections, as depicted in Figure 12. The articulating module was made to face each divided section. The endoscope camera at the tip of the articulating module captured images of each octant and confirmed that the text on the sections was correctly recognized. Figures 12B and C show the position of the articulating module and images taken in sections ④ and ⑤. Figure 12D shows an example trajectory of the tip of the continuum body in a trial. In the trial, the tip moved from a starting point through section ⑦, middle section, section ③, middle section, and section ④, to middle section by a user’s input of the smartphone app.

### Phantom Imaging

**Figure 13:**
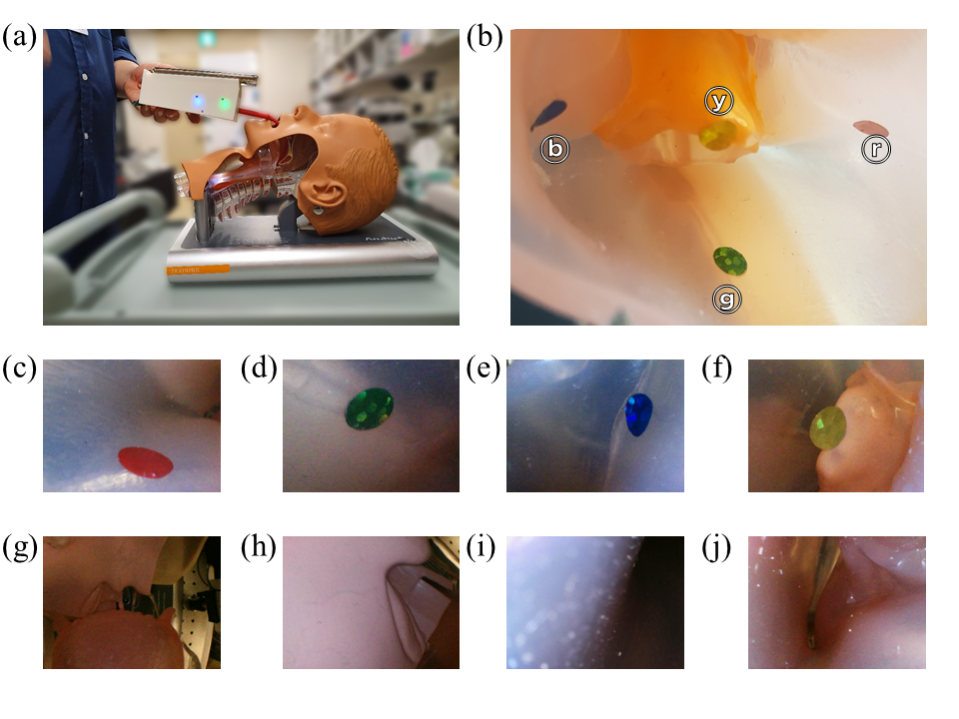
Articulation experiment on the laryngopharyngeal phantom: (a) endoscope experiment in operation, (b) placement of stickers attached as indices in the oropharynx area of the phantom (upper side, yellow ⓨ; on the left, blue ⓑ; on the right, red ⓡ; and on the lower side, green ⓖ sticker), (c-f) images of the stickers obtained in the articulation mode, and (g-j) images captured by the camera, going deep from the oral cavity to the oropharynx.

To verify the ability to steer the articulating module, we placed 4 colored stickers (ventral, dorsal, left, and right) on the inner side of each of two human anatomical phantoms and captured images of the marked locations following a simulated clinical endoscope insertion. Figure 13A depicts the endoscope during the experiment. The operator was able to successfully insert the endoscope into a laryngopharyngeal phantom using only one hand. The placement of the colored marks is shown in Figure 13B. The marks were in the oropharynx area: yellow, on the epiglottis; green, on the back wall of the oropharynx; and red and blue, on the right and left walls of the oropharynx, respectively. After the endoscope insertion, it was possible to image each of the marks by controlling the articulating module to face in the desired direction. Figures 13C-F present the resulting images. All 4 marks were clearly observed and within the focal range of the endoscope. Finally, we captured images while inserting the endoscope deeper into the phantom. Figures 13G-J show the insertion path through the laryngopharyngeal phantom.

**Figure 14:**
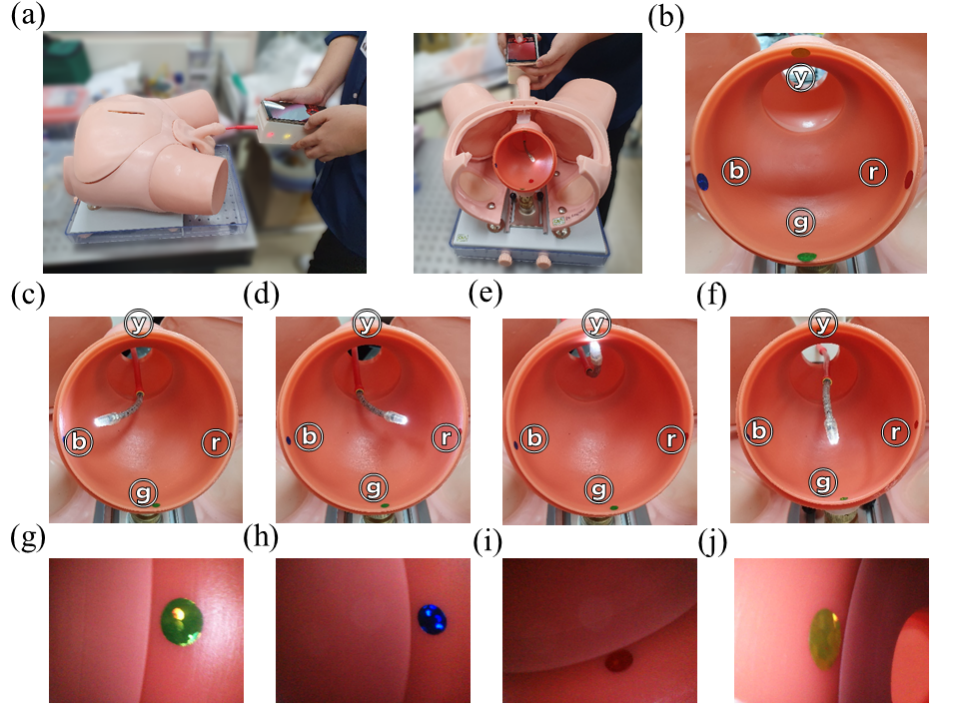
Articulation experiment on the bladder phantom: (a) endoscope experiment in operation, (b) placement of stickers on the inner side of the phantom (upper side, yellow ⓨ; on the left, blue ⓑ; on the right, red ⓡ; on the lower side, green ⓖ sticker, which was attached as an index), (c-f) articulation in four directions, and (g-j) corresponding images of the stickers obtained using the homebuilt smartphone app.

A similar experiment simulated steering of the articulating module into and around the bladder. As before, 4 directions on the inner side of a bladder phantom were marked with colored adhesives. The endoscope was inserted into the bladder phantom via a simulated male urinary tract by an operator using one hand for stability and the other hand for insertion and steering, as shown in Figure 14A. The arrangement of the marks is shown in Figure 14B. The articulating module of the endoscope was able to face each marking successfully without movement at the proximal end of the endoscope module, as shown in Figures 14C-F. The successfully captured images of each of the four marks are shown in Figures 14G-J. The results, although captured on a phantom, indicate that the present rigid cystoscopy procedures can remain successful and be made significantly less invasive with the use of a similar articulating flexible endoscope design.

## Discussion

### Principal Findings

A portable wide-field endoscope combined with a smartphone was successfully developed. Using an app programmed in smartphone, an actively controlled articulable endoscope was demonstrated. Our device is compact in design, with a width and length similar to those of a smartphone. The endoscope system combined with smartphone can fit into one hand of either a male or a female operator. The weight of our device is 309 g without a smartphone. By attaching a smartphone, the weight can vary from 400 g to 500 g, depending on the conventional smartphone model. Therefore, as demonstrated experimentally, the user may freely conduct endoscopic procedures without being restricted to medical facilities such as clinics and hospitals. However, it is not limited to clinical applications but could be adopted for preclinical, veterinary, and industrial applications.

To facilitate the clinical use of the device, several human engineering factors were considered when implementing the UI design. The UI employs a joystick-like controller, and 4 fine control buttons are placed in the four corresponding directions, facilitating both intuitive and precise control, satisfying clinical needs. Placement of the additional buttons is low on the smartphone, allowing for ambidextrous control such that users may operate the virtual joystick, movie/still-image capture buttons, brightness adjustment, and contrast adjustment with ease even while focused on steering the endoscope.

The device runs on a smartphone, which is a highly available Linux computer, and the app for the device was developed in the C and Java languages, which are freely available. Because the application code is based on open-source projects, it can be easily modified by companies, local authorities, or other researchers for adding functionality. The Open Source Computer Vision Library (OpenCV) library is an open-source BSD-licensed library that includes hundreds of computer-vision algorithms [27] and provides programmatical brightness and contrast adjustment in app modules. Usually, brightness and contrast are the most significant factors that contribute to visual recognition; therefore, we made the contrast and brightness user-adjustable by adding postprocessing capabilities to the smartphone-based app. Because the app is based on OpenCV open source image processing program, complicated algorithms that can help diagnosis can be implemented. For example, OpenCV can provide the basic level of motion recognition, contour recognition, and background removal. Combined with the versatility and rapid processing ability of a smartphone, we can implement fairly complicated algorithms that generally require postprocessing on a desktop computer. Furthermore, the processed images are saved in the phone’s internal memory and can be easily accessed by other apps for further analysis or mobile sharing with other caregivers. This provides a new scheme of mobile examination and telemedicine. In industrial applications or at home, the user can easily send pictures to experts to examine data. In medical applications, endoscopes can be operated by trained users outside the hospital, and images can be shared with physicians and specialists for examination in real time. In addition, it enables easy dissemination of endoscopy devices in areas where medical benefits are insufficient.

The articulating module of the endoscope can be articulated in 2 DOFs. The actively controlled vertical and horizontal articulation of the articulating module make it easier to navigate through complex turns compared with conventional flexible endoscopes. This allows the user to easily find the desired location in a narrow area. To examine the feasibility of applying our articulating endoscope to medical examination, we used laryngopharyngeal and bladder phantoms. The pharynx and larynx are connected to the oropharyngeal area, which is distinguished by the epiglottis. The oropharynx and nasopharynx regions are smoothly connected to the pharynx but curve sharply when entering the larynx. Therefore, it is more difficult for the user to access the larynx when nonarticulating endoscopes are employed. As mentioned in the Introduction, patients with strong gag reflexes or limited jaw and neck movements are more likely to reject endoscopes when the endoscope touches the wall of the laryngopharyngeal complex. By using articulable endoscopes, the contact of endoscope tips with sensitive regions of the inner wall can be reduced. The depth this endoscopic device can reach depends on the length of each body shown in Figure 3. Each part in the endoscopic module body has the following dimensions: length of 52 mm for the distal, 150 mm for the flexible section, and 52 mm for the rigid section. Thus, the total length is approximately 254 mm. The average length of an adult’s vocal cords, from mouth to vocal cords, is approximately 160 mm according to Cherng et al [28]. Therefore, the length of the developed device is sufficient for laryngopharyngeal applications. Furthermore, the length of conventional rigid resectoscopes for the transurethral resection of bladder tumors is 150 mm; therefore, the developed device can be employed for bladder diagnostic applications.

In addition to improving endoscopic access, the movability of the distal end of the endoscope significantly increases the range of visibility. For example, it is necessary to move the proximal part of both rigid and flexible cystoscopes if imaging on the opposite face of the bladder is desired. This movement can cause problems, including patient discomfort and destruction of the urethra. With the articulation, the endoscope unit attached to the distal articulating module can be articulated to face the desired direction without moving the proximal part of endoscope. The transparent sphere and bladder phantom experiments demonstrated this advantage of integrating the continuum body with the smartphone-attached endoscope. In the experiments, we succeeded in imaging all octants of the transparent sphere and all 4 marks on the bladder phantom without moving the proximal rigid part of the endoscope module. Thus, our device can face the desired part of the inner side of the bladder and obtain images [11].

The potential of portable smartphone-integrated medical devices involving endoscopes attached to the built-in camera of a smartphone has been discussed [29,30]. Using both rigid and flexible endoscopes, images are successfully captured [29]. Although their endoscope system is easy to manufacture, portable, and cheaper than conventional one, the device proposed in this study has several advantages over the devices of Bae et al [29]. First, our system uses an USB OTG connection, which allows the user to access external devices such as higher functions of cameras, data reader, or tablet computer, etc. This frees the endoscope from the limitations of internal smartphone cameras and from the other optical adjustments needed for magnification and precise alignment of the optical path. Moreover, since the performance of the external camera is independent from the specifications of the smartphone, as long as the device supports OTG connection, the external camera provides stable performance. Second, the proposed device is articulable and actively controlled, and thus allows imaging in deeper and from more complex organ structures compared with nonarticulable devices. This means that images of deeper positions can be obtained in a safe and stable state. Besides, when combined with biopsy devices, the procedures for biopsies and diagnosis in more difficult locations are easier. Third, depending on the capabilities of the mobile apps, it can be extended to a variety of application software such as image processing application, artificial intelligence video analysis application, statistical extraction application, and big data analysis application. By linking together, it is possible to add more and different technologies, thus overcoming the technical limitations of existing endoscopes.

Battery-based systems help users move freely out of a fixed area, independent from power generators. The proposed articulable device attached to a smartphone can be operated by a single operator outside the hospital and can be powered entirely by its own battery, isolated from the electrical grid. Since the battery is independent from the power generator, if the power runs out, it can sustain the system. The mobility of the device allows the user to employ the endoscope outside well-developed areas and makes it more suitable for use in underdeveloped countries, where electrical supplies are more likely to be interrupted due to power shortages, political complications, or poverty [31,32]. Moreover, portability may allow care providers to bring health services to rural areas and perform simple endoscopy for patients in underdeveloped areas. The smartphone allows visiting doctors or physicians to perform simple endoscopy, send images to faraway specialists for analysis, and then make appropriate treatment decisions.

The device is used for tubular organs and naturally opened orifices such as superficial layers of respiratory tract, urinary bladder, urethra, vaginal tract, and proximal part of colon to monitor lesion, polyps, stones, cancerous tissue, and abnormal dysplasia. Target users are medical professional, since nonprofessionals cannot keep up with medical knowledge or sterilization process, particularly in developing countries. Moreover, several countries have laws forbidding medical examination by nonprofessionals. Due to general medical guidelines, the endoscope probe must be sterilized before it is used and replaced every 20 rounds of examination session.

Finally, the portable articulable endoscope is far cheaper than a conventional endoscope system; the price to manufacture it is approximately US $480, excluding the smartphone. The estimated price will decrease with mass production. Table 2 compared available solutions that are being sold on the market. The price of nonarticulating endoscopes is considerably cheap; however, they are not articulable, have low image quality, and have no programmatic functions that help diagnosis. In contrast, conventional gastrointestinal endoscope systems have considerably more functions; however, they cannot implement complex algorithms such as machine learning or be taken out of the hospital facilities; further, they are very expensive. Therefore, as long as therapeutic accessories are not necessary, our system can be a simple and affordable solution for rapid diagnosis in developing countries.

**Table 2:** Comparison of available options.

Options	Smartphone-connected nonarticulating endoscope	Smartphone-based articulable endoscope, (proposed system)	Conventional gastrointestinal endoscope
Price (w/o smartphone), US$	10-100	473	20,000-120,000
Price (w/ smartphone), US$	300-1100 depending on smartphone model	700-1500 depending on smartphone model	N/A^a^
Probe tip DOF^b^	none	2 DOF	2 DOF

^a^N/A: not applicable.

^b^DOF: degree of freedom.

Considering that conventional endoscope systems from major companies and including camera, light source, etc, are usually priced above US $50,000 [29,33], the proposed device reduces the cost of care and increases access for people in underfunded and underdeveloped medical systems. With the acceptance of medical practitioners and up-to-date image processing algorithm, we could confirm that the proposed system has ample potential for use as a point-of-care diagnosis in developing countries.

### Limitations

Since the developed system is a handheld type, movement of the endoscope probe may occur somewhat compared with the conventional mounted endoscope system. There is also a risk of sterilization and replacement of the endoscope probe, and there is a risk of breaking the drive wire if tension is applied that exceeds the operating range of the device. However, these sterilization, cleaning and wire breakage issues can be solved through simple replacement by making the endoscope probe into a module.

### Conclusion

We designed, fabricated, and tested a portable smartphone-based and actively controlled articulable endoscope system. Microprocessors and smartphones have advanced to the point where they can serve as small computers. These portable devices open a new possibility for portable, cost-effective, and easy-to-operate at-home medical devices. In this study, we introduced a low-cost articulable endoscope system for mobile health care with an external camera as image sensor and smartphone as controller, providing advantages such as articulation, image control, mobility, and communication. We evaluated the feasibility of this system via phantom imaging. The results indicated that the device and homebuilt app have ample potential for preclinical, clinical, veterinary, and industrial applications. Specifically, the proposed endoscope could be a powerful tool for health care providers in regions with limited health care service.

## Abbreviations

3D: three-dimensional
CMOS: complementary metal-oxide semiconductor
DOF: degree of freedom
Open CV: Open Source Computer Vision Library
OTG: on-the-go
UI: user interface
